# Association between weekend catch-up sleep and dyslipidemia among Korean workers

**DOI:** 10.1038/s41598-023-28142-w

**Published:** 2023-01-17

**Authors:** Ye Seul Jang, Yu Shin Park, Kyungduk Hurh, Eun-Cheol Park, Sung-In Jang

**Affiliations:** 1 Department of Public Health, Graduate School, Yonsei University, Seoul, Republic of Korea; 2 Institute of Health Services Research, Yonsei University, Seoul, Republic of Korea; 3 Department of Preventive Medicine and Institute of Health Services Research, Yonsei University College of Medicine, 50 Yonsei-Ro, Seodaemun-Gu, Seoul, 03722 Republic of Korea

**Keywords:** Cardiology, Health care

## Abstract

Within competitive sociocultural environments, most Korean workers are likely to shorten their sleep duration during the weekday. Short sleep duration is associated with dyslipidemia; however, studies on the correlation between various sleep patterns and dyslipidemia are still lacking. In hence this study aimed to investigate the association between weekend catch-up sleep (CUS) and dyslipidemia among South Korean workers. Our study used data from the 8th Korea National Health and Nutrition Examination Survey (KNHANES). The analysis covered 4,085 participants, excluding those who were diagnosed with dyslipidemia and not currently participating in economic activities. Weekend CUS was calculated as the absolute difference between self-reported weekday and weekend sleep duration. Dyslipidemia was diagnosed based on the levels of total cholesterol, high-density lipoprotein (HDL) cholesterol, low-density lipoprotein (LDL) cholesterol, and triglycerides in blood samples collected after 9–12 h of fasting. After adjusting for sociodemographic, economic, health-related, and sleep-related factors, a negative association of weekend CUS with dyslipidemia was observed in male workers (odds ratio: 0.76, 95% confidence interval: 0.61–0.95). Further, workers with total sleep duration of 7–8 h, night workers, and white-collar workers with CUS were at relatively low risk of dyslipidemia compared to the non-CUS group. Less than 2 h of weekend CUS was negatively related to dyslipidemia in Korean workers, especially males. This suggests that sleeping more on weekends for workers who had a lack of sleep during the week can help prevent dyslipidemia.

## Introduction

Cardiovascular disease (CVD) is the cause of substantial social burdens worldwide and is the leading cause of death in South Korea, where the CVD-associated mortality rate has been gradually increasing recently^1^. Dyslipidemia, a major risk factor for CVD, is increasing in prevalence in South Korea^1,2^. Over the past few decades, various lifestyle changes have increased the prevalence of dyslipidemia^3^. Many studies have reported several risk factors for dyslipidemia, such as age, hypertension, and cigarette smoking^4^. As other risk factors of dyslipidemia are being reduced or controlled better than ever before, negative changes in lifestyle patterns, such as lack of exercise, excessive alcohol intakes or else, might be responsible^1,5^.

Sleep duration is an important part of a healthy lifestyle, and insufficient sleep is one of the most common sleep-related problems^6^. However, excessive sleep is also associated with worsening health status. Therefore, the importance of an optimal duration and quality of sleep has been recognized^7^. The international classification of sleep disorders notes that the optimal sleep duration is 7–8 h^8^.

In the modern age, sleep restriction often occurs for social requirements or work schedules, with a trend toward reduced sleep duration. Workers who live in an environment with a lack of sufficient sleep on weekdays due to work schedules or other causes often sleep more on weekends, which is known as weekend catch-up sleep (CUS). Weekend CUS is calculated as the absolute difference between the weekday and weekend sleep duration^9^.

Most workers make up for their short weekday sleep with extended weekend sleep^10^. According to previous studies, catching up on sleep on weekends appears to limit the comorbid risks associated with sleep debt^11^. Short sleep duration is associated with dyslipidemia^12^, but studies on the correlation between the various patterns of sleep and dyslipidemia are still lacking. Hence, this study aimed to investigate the association between weekend CUS and dyslipidemia among Korean workers using a nationally representative sample of Korea. We hypothesized that making up for sleep over the weekend would be associated with a lower risk of dyslipidemia. We also identified the relationship between dyslipidemia according to the difference in CUS through subgroup analysis.

## Methods

### Data

The study data were obtained from the 2019 and 2020 Korean National Health and Nutrition Examination Survey (KNHANES). The KNHANES is a cross-sectional nationwide survey and is conducted by the Korean Center for Disease Control and Prevention^13^. The KNHANES provides a nationally representative sample of the South Korean population residing in Korea, using a complex and multistage clustered probability design.

### Participants

The current study used data from the 2019 and 2020 KNHANES, which contains data from 15,469 participants. Participants < 19 years of age (n = 2,730) were excluded from this study. As we aimed to analyze workers, we also excluded individuals not currently participating in economic activities (n = 5,371). In addition, those who were diagnosed with dyslipidemia and current is currently undergoing treatments (n = 1,207) or had missing data (n = 2,076) were excluded. Finally, the study comprised of 4,085 participants (2,206 males and 1,879 females). This study did not require prior consent or approval from an Institutional Review Board because the KNHANES is a secondary dataset and consists of already de-identified data available in the public domain.

### Variables

The main variable of interest was weekend CUS calculated using the average weekday and weekend sleep duration from the relevant KNHANES questionnaire. Participants’ average weekday and weekend sleep durations were calculated based on their responses to the following questions: On a weekday (or working day), at How many hours do you usually sleep a day? On a weekend (or the day when you do not work, the day before you do not work), How many hours do you usually sleep a day? Weekend CUS was defined as sleep duration in the weekend being longer than that in weekdays^14^ Weekend CUS was calculated as the average weekend sleep duration minus the average weekday sleep duration.. Participants were then divided into non-CUS (≤ 0 h) and CUS (0 > h) groups^10^. Additionally, we classified CUS duration into 0 < to 1, 1 < to 2, and > 2 h for subgroup analysis.

The dependent variable was the prevalence of dyslipidemia diagnosed based on the levels of total cholesterol, high-density lipoprotein (HDL) cholesterol, low-density lipoprotein (LDL) cholesterol, and triglycerides in blood samples collected after 9–12 h of fasting. According to the 2018 Korean Guidelines for the Management of Dyslipidemia, for diagnosis of dyslipidemia, one of the following four criteria was required: (1) total cholesterol ≥ 240 mg/dL, (2) HDL cholesterol ≤ 40 mg/dL, (3) LDL cholesterol ≥ 160 mg/dL, or (4) triglycerides ≥ 200 mg/dL^15^.

The following covariates were included in the analyses: The sociodemographic factors were age (19–29, 30–39, 40–49, 50–59, and ≥ 60 years) and sex (male and female). The socioeconomic factors were education level (middle school or lower, high school, or university or higher), region (metropolitan or rural area), marital status (married or unmarried), occupation (white collar, pink collar, blue collar), and household income (high, middle-high, middle-low, or low). The health-related factors were obstructive sleep apnea calculated by STOP-bang (yes or no), alcohol consumption status (less 1 time per month, 2–4 times per months, over 2 times per week) and smoking status (yes or no). In addition, adjustments were made for average total sleep duration (< 7,7–8,8 <), work pattern (day, night, shift work), physical activity (yes or no), body mass index (underweight, normal, overweight), menopause status (yes or no), hypertension (yes or pre-hypertension or no), and diabetes (yes or prediabetes or no).

### Statistical analyses

Owing to sex differences in physical conditions, all analyses were stratified by sex^16^. Descriptive analysis using by chi-square test was performed to examine the distribution of the general characteristics of the study population. Multiple logistic regression modelling was used to assess the association between CUS and prevalence of dyslipidemia after adjusting for all covariates. In addition, to find out the association according to the subdivided categories of weekend CUS and dyslipidemia, multiple logistic regression analyses of subgroups were also performed. ORs and 95% CIs were calculated to compare the data of participants with dyslipidemia. Variables were clustered, stratified, and weighted to account for the limited proportion of participants retained in the final analysis^17^. SAS (version 9.4M6; SAS Institute, Cary, NC) was used for all statistical analyses.

## Results

Table 1 summarizes the general characteristics of the study population, stratified by sex. Of the 4,085 participants, 2,206 were males and 1,879 were females. Of these, 1,290 individuals (881 males and 409 females) had dyslipidemia. The prevalence of dyslipidemia was greater among non-CUS workers compared to those who had weekend CUS (non-CUS: 544/1,267, 42.9%; CUS: 337/939, 35.9%). A similar trend was observed among females (non-CUS: 253/1,036, 24.4%; CUS: 156/843, 18.5%).

**Table 1:** General characteristics of the study population.

Variables	Dyslipidemia^a^													
	Male							Female						
	Total		No		Yes		*P*-value	Total		No		Yes		*P*-value
	N	%	N	%	N	%		N	%	N	%	N	%	
Total (*N* = 4,085)	2,206	100.0	1,325	60.1	881	39.9		1,879	100.0	1,470	78.2	409	21.8	
Weekend catch up sleep							0.001							0.002
Non-CUS (≤ 0)	1,267	57.4	723	57.1	544	42.9		1,036	55.1	783	75.6	253	24.4	
CUS	939	42.6	602	64.1	337	35.9		843	44.9	687	81.5	156	18.5	
Age							< 0.0001							< 0.0001
19–29	289	13.1	211	73.0	78	27.0		303	16.1	279	92.1	24	7.9	
30–39	477	21.6	293	61.4	184	38.6		385	20.5	324	84.2	61	15.8	
40–49	516	23.4	275	53.3	241	46.7		524	27.9	439	83.8	85	16.2	
50–59	456	20.7	257	56.4	199	43.6		407	21.7	264	64.9	143	35.1	
60 ≤	468	21.2	289	61.8	179	38.2		260	13.8	164	63.1	96	36.9	
Total sleep duration(hours)							0.290							0.254
< 7	779	35.3	451	57.9	328	42.1		575	30.6	454	79.0	121	21.0	
7–8	1,186	53.8	725	61.1	461	38.9		986	52.5	761	77.2	225	22.8	
8 <	241	10.9	149	61.8	92	38.2		318	16.9	255	80.2	63	19.8	
Obstructive sleep apnea^a^							0.289							0.598
Yes	2,194	99.5	1,316	60.0	878	40.0		1,878	99.9	1,469	78.2	409	21.8	
No	12	0.5	9	75.0	3	25.0		1	0.1	1	100.0	0	0.0	
Work pattern							0.402							0.332
Day	1,834	83.1	1,091	59.5	743	40.5		1,567	83.4	1,229	78.4	338	21.6	
Night	220	10.0	141	64.1	79	35.9		255	13.6	193	75.7	62	24.3	
Shift work	152	6.9	93	61.2	59	38.8		57	3.0	48	84.2	9	15.8	
Income							< 0.0001							0.003
Low	177	8.0	116	65.5	61	34.5		164	8.7	115	70.1	49	29.9	
Middle low	493	22.3	281	57.0	212	43.0		401	21.3	299	74.6	102	25.4	
Middle high	686	31.1	415	60.5	271	39.5		579	30.8	458	79.1	121	20.9	
High	850	38.5	513	60.4	337	39.6		735	39.1	598	81.4	137	18.6	
Region							0.648							0.493
Urban	981	44.5	584	59.5	397	40.5		855	45.5	675	78.9	180	21.1	
Rural	1,225	55.5	741	60.5	484	39.5		1,024	54.5	795	77.6	229	22.4	
Occupation							< 0.0001							0.005
White collar	932	42.2	546	58.6	386	41.4		960	51.1	780	81.3	180	18.8	
Pink collar	348	15.8	216	62.1	132	37.9		513	27.3	387	75.4	126	24.6	
Blue collar	926	42.0	563	60.8	363	39.2		406	21.6	303	74.6	103	25.4	
Smoking							0.047							0.480
Yes	1,481	67.1	911	61.5	570	38.5		1,764	93.9	1,377	78.1	387	21.9	
No	725	32.9	414	57.1	311	42.9		115	6.1	93	80.9	22	19.1	
Drinking							0.166							0.006
Less 1 time per month	805	36.5	463	57.5	342	42.5		1,067	56.8	807	75.6	260	24.4	
2–4 times per month	636	28.8	395	62.1	241	37.9		512	27.2	414	80.9	98	19.1	
Over 2 times per week	765	34.7	467	61.0	298	39.0		300	16.0	249	83.0	51	17.0	
Physical activity							0.004							0.184
Active	1,143	51.8	653	57.1	490	42.9		1,090	58.0	841	77.2	249	22.8	
Inactive	1,063	48.2	672	63.2	391	36.8		789	42.0	629	79.7	160	20.3	
BMI							< 0.0001							< .0001
Underweight	642	29.1	486	75.7	156	24.3		1,058	56.3	913	86.3	145	13.7	
Normal	578	26.2	349	60.4	229	39.6		354	18.8	256	72.3	98	27.7	
Overweight	986	44.7	490	49.7	496	50.3		467	24.9	301	64.5	166	35.5	
Hypertension							< 0.0001							< .0001
No	850	38.5	581	68.4	269	31.6		1,151	61.3	962	83.6	189	16.4	
Pre-Hypertension	773	35.0	439	56.8	334	43.2		426	22.7	319	74.9	107	25.1	
Hypertension	583	26.4	305	52.3	278	47.7		302	16.1	189	62.6	113	37.4	
Diabetes							< 0.0001							< .0001
No	977	44.3	661	67.7	316	32.3		1,099	58.5	951	86.5	148	13.5	
Pre-Diabetes	981	44.5	554	56.5	427	43.5		689	36.7	472	68.5	217	31.5	
Diabetes	248	11.2	110	44.4	138	55.6		91	4.8	47	51.6	44	48.4	
Menopause														< .0001
No								1,341	71.4	1,118	83.4	223	16.6	
Yes								538	28.6	352	65.4	186	34.6	
Year							0.062							0.652
2019	1,188	53.9	735	61.9	453	38.1		1,029	54.8	801	77.8	228	22.2	
2020	1,018	46.1	590	58.0	428	42.0		850	45.2	669	78.7	181	21.3	

*BMI* body mass index.

^a^One of the following four criteria was required: (1) total cholesterol ≥ 240 mg/dL, (2) HDL cholesterol ≤ 40 mg/dL, (3) LDL cholesterol ≥ 160 mg/dL, or (4) triglycerides ≥ 200 mg/dL.

^b^Only for over 40 years of age.

Table 2 presents the results from the multiple logistic regression analysis of the association between CUS and dyslipidemia. There was a significant association in males between weekend CUS and dyslipidemia (odds ratio [OR]: 0.76, 95% confidence interval [CI]: 0.61–0.95). However, no such association was found for females.

**Table 2:** Association between *Dyslipidemia* and subject demographic.

Variables	Male		Female	
	Dyslipidemia^a^		Dyslipidemia	
	OR	95% CI	OR	95% CI
Weekend catch up sleep				
Non-CUS (≤ 0)	1.00			1.00
CUS	0.76	(0.61–0.95)	0.86	(0.62–1.19)
Age				
19–29	1.00		1.00	
30–39	1.46	(0.99–2.15)	2.85	(1.52–5.36)
40–49	2.06	(1.40–3.01)	2.31	(1.25–4.26)
50–59	1.68	(1.14–2.47)	5.86	(2.82–12.17)
60 ≤	1.24	(0.78–1.98)	5.94	(2.49–14.18)
Total sleep duration(hours)				
< 7	0.98	(0.78–1.23)	0.61	(0.45–0.83)
7–8	1.00			1.00
8 <	1.21	(0.84–1.76)	1.06	(0.70–1.61)
Obstructive sleep apnea^b^				
Yes	0.33	(0.07–1.46)	–	–
No	1.00		1.00	
Work pattern				
Day	1.00		1.00	
Night	0.77	(0.53–1.13)	1.07	(0.71–1.62)
Shift work	1.26	(0.83–1.91)	0.55	(0.28–1.09)
Income				
Low	0.96	(0.57–1.61)	1.33	(0.75–2.35)
Middle low	1.24	(0.94–1.62)	1.29	(0.88–1.89)
Middle high	0.99	(0.77–1.27)	0.96	(0.68–1.35)
High	1.00		1.00	
Region				
Urban	1.00		1.00	
Rural	0.92	(0.75–1.14)	1.12	(0.83–1.51)
Occupation				
White collar	1.00		1.00	
Pink collar	0.98	(0.71–1.36)	0.87	(0.60–1.26)
Blue collar	0.86	(0.67–1.09)	0.52	(0.36–0.77)
Smoking				
Yes	1.41	(1.12–1.77)	0.98	(0.53–1.80)
No	1.00		1.00	
Drinking				
Less 1 time per month	1.00		1.00	
2–4 times per month	0.83	(0.64–1.07)	1.04	(0.75–1.44)
Over 2 times per week	0.72	(0.57–0.92)	0.66	(0.44–0.99)
Physical activity				
Active	1.00		1.00	
Inactive	1.08	(0.87–1.35)	0.88	(0.65–1.18)
BMI				
Underweight	0.52	(0.40–0.69)	0.58	(0.41–0.81)
Normal	1.00		1.00	
Overweight	1.50	(1.17–1.91)	1.63	(1.12–2.36)
Hypertension				
No	1.00		1.00	
Pre-Hypertension	1.312	(1.04–1.66)	1.062	(0.75–1.51)
Hypertension	1.44	(1.07–1.95)	1.34	(0.89–2.02)
Diabetes				
No	1.00		1.00	
Pre-Diabetes	1.40	(1.13–1.74)	1.68	(1.20–2.36)
Diabetes	2.14	(1.47–3.11)	2.18	(1.14–4.18)
Menopause				
No			1.00	
Yes			1.07	(0.68–1.67)
Year				
2019			1.00	
2020	1.17	(0.96–1.44)	1.01	(0.75–1.35)

*BMI* body mass index.

^a^One of the following four criteria was required: (1) total cholesterol ≥ 240 mg/dL, (2) HDL cholesterol ≤ 40 mg/dL, (3) LDL cholesterol ≥ 160 mg/dL, or (4) triglycerides ≥ 200 mg/dL.

^b^Only for over 40 years of age.

The results of the subgroup analysis stratified by total sleep duration, work pattern, and occupational categories are shown in Table 3. Male CUS workers who slept for a total average of 7–8 h were less likely to have dyslipidemia compared to non-CUS workers (OR: 0.70, 95% CI: 0.52–0.94). Similarly, male CUS workers with white-collar jobs were at less risk of dyslipidemia compared to non-CUS workers (OR: 0.68, 95% CI: 0.49–0.94). Regardless of sex, night workers with CUS showed a significant association between weekend CUS and dyslipidemia compared to those without CUS (male: OR: 0.38, 95% CI: 0.18–0.83, female: OR: 0.30, 95% CI: 0.13–0.73).

**Table 3:** Results of subgroup analysis stratified by independent variables.

	Male			Female		
	Dyslipidemia^a^					
	Catch up sleep			Catch up sleep		
	Non-CUS		CUS	Non-CUS		CUS
	OR	OR	95% CI	OR	OR	95% CI
Age						
19–29	1.00	0.56	(0.27–1.17)	1.00	1.40	(0.55–3.57)
30–39	1.00	0.62	(0.39–0.99)	1.00	0.73	(0.37–1.45)
40–49	1.00	0.98	(0.65–1.48)	1.00	0.54	(0.28–1.05)
50–59	1.00	0.70	(0.44–1.13)	1.00	1.03	(0.58–1.81)
60 ≤	1.00	0.95	(0.50–1.80)	1.00	1.90	(0.68–5.31)
Total sleep duration(hours)						
< 7	1.00	0.76	(0.52–1.10)	1.00	0.89	(0.49–1.60)
7–8	1.00	0.70	(0.52–0.94)	1.00	0.85	(0.56–1.27)
8 <	1.00	1.61	(0.64–4.07)	1.00	0.78	(0.30–1.99)
Obstructive sleep apnea^b^						
Yes	1.00	0.75	(0.60–0.94)	1.00	0.86	(0.62–1.19)
No	1.00	–	–	1.00	–	–
Work pattern						
Day	1.00	0.79	(0.62–1.01)	1.00	0.97	(0.68–1.37)
Night	1.00	0.38	(0.18–0.83)	1.00	0.30	(0.13–0.73)
Shift work	1.00	0.72	(0.26–1.98)	1.00	–	–
Occupation						
White collar	1.00	0.68	(0.49–0.94)	1.00	0.97	(0.60–1.57)
Pink collar	1.00	0.78	(0.43–1.41)	1.00	0.61	(0.30–1.24)
Blue collar	1.00	0.76	(0.53–1.10)	1.00	0.99	(0.49–2.01)
Hypertension						
No	1.00	0.64	(0.45–0.91)	1.00	1.17	(0.73–1.86)
Pre-Hypertension	1.00	0.86	(0.60–1.24)	1.00	0.56	(0.28–1.13)
Hypertension	1.00	0.82	(0.51–1.31)	1.00	0.58	(0.27–1.23)
Diabetes						
No	1.00	1.31	(0.56–1.09)	1.00	1.07	(0.66–1.74)
Pre-Diabetes	1.00	0.78	(0.55–1.11)	1.00	0.64	(0.40–1.04)
Diabetes	1.00	0.58	(0.28–1.20)	1.00	4.09	(0.87–19.35)

^a^One of the following four criteria was required: (1) total cholesterol ≥ 240 mg/dL, (2) HDL cholesterol ≤ 40 mg/dL, (3) LDL cholesterol ≥ 160 mg/dL, or (4) triglycerides ≥ 200 mg/dL.

^b^Only for over 40 years of age.

Table 4 shows the results of subgroup analysis stratified by classified CUS. Males who had ≤ 2 h of CUS were significantly less likely to have dyslipidemia (0 < CUS ≤ 1: OR: 0.74, 95% CI: 0.55–0.998, 1 < CUS ≤ 2: OR: 0.64, 95% CI: 0.47–0.89). This association was not observed in females.

**Table 4:** Result of interesting subgroup analysis according to Catch up sleep level.

Male	Dyslipidemia^a^			
	Male		Female	
	OR	95% CI	OR	95% CI
Weekend catch up sleep				
Non-CUS (≤ 0)	1.00		1.00	
0 < CUS ≤ 1	0.74	(0.55–1.00)	0.87	(0.53–1.43)
1 < CUS ≤ 2	0.64	(0.47–0.89)	0.83	(0.56–1.24)
CUS > 2	1.04	(0.73–1.47)	0.88	(0.55–1.41)

^a^One of the following four criteria was required: (1) total cholesterol ≥ 240 mg/dL, (2) HDL cholesterol ≤ 40 mg/dL, (3) LDL cholesterol ≥ 160 mg/dL, or (4) triglycerides ≥ 200 mg/dL.

## Discussion

In this study, we found that Korean male workers with ≤ 2 h of CUS had a decreased risk of dyslipidemia compared to those without CUS after adjusting for potential covariates. Further, workers with a total sleep duration of 7–8 h, night workers, and white-collar workers with CUS were at relatively low risk of dyslipidemia compared with those without CUS.

Sleep is an important factor in healthcare^18^. Reduced sleep quality or sleep duration could be risk factors for poor physical and psychological health^19,20^. Other studies have shown that excessive sleep has adverse effects on health outcomes^21^. Optimal sleep management is essential for healthcare, but most Koreans, especially those who work, do not get enough sleep^22,23^. Although most people have different lifestyles, Korean workers tend to make up for their lack of sleep on weekdays with weekend sleep^24^. According to studies, to cope with weekly sleep deprivation, weekend CUS is undertaken, which is associated with a lower prevalence of hypertension, obesity, and serum high-sensitivity C-reactive protein levels^25–27^. A previous epidemiological study reported that insufficient sleep duration increases the risk of CVD^28^. Likewise, sufficient sleep can reduce the risk of developing CVD^29^. This may explain our finding that supplementing insufficient sleep with weekend CUS is linked to a reduced risk of dyslipidemia.

In our study, workers who had CUS and an optimal sleep duration (7–8 h) on weekdays had a negative relationship with dyslipidemia compared to those who had abnormal sleep durations of < 7 h or > 8 h. A previous study has suggested that abnormal sleep duration during the week is associated with increased mortality in individuals < 65 years old^20^. Similarly, another study showed that those who had appropriate sleep with CUS had a negative correlation with obesity^30^. Therefore, our study suggests the need to keep an optimal sleep duration even if it is supplemented on weekends.

Night work is more strongly associated with dyslipidemia, compared to day or other shift work^31^. Night workers usually receive less sleep than day workers^32^. Sleep deprivation negatively affects metabolism and promotes the development of an atherogenic lipid profile^33^. This may explain our finding that night workers’ sleep supplementation on the weekend showed a negative relationship with dyslipidemia compared to day or shift workers. Furthermore, the risk of dyslipidemia is lower when there is ≤ 2 h difference in sleep time between weekdays and weekends. On the other hand, those with > 2 h difference showed a positive relationship, but it was not statistically significant. Obviously, insufficient sleep is associated with negative health effects; however, habitual excessive sleep can also increase the risk of mortality, and if the degree of misalignment is severe, the compensatory effect might disappear^34,35^. Hence, to protect workers from dyslipidemia, we need to identify how to attain enough sleep in general and achieve a balanced sleep duration between weekdays and weekends.

Although the results of this study serve as further evidence in clarifying the negative association between weekend CUS and dyslipidemia, especially among Korean male workers, it has some limitation. First, this study used a cross-sectional data set; thus, we could only determine the association and not investigate the causal relationship between those variables. Therefore, additional research is needed to infer an accurate causality. Second, data regarding sleep time comes from self-report questionnaires; inaccuracies may, thus, occur. As such, the possibility of a difference between actual and reported sleep time cannot be excluded. Third, due to the data limitation, potential risk factors related to sleep and dyslipidemia may existed, such as a diagnosis of insomnia or other medications which affects the levels of lipids not considered in this study.

Despite these limitations, this study has also several strengths. First, dyslipidemia was measured through clinical testing; hence, it was based on more reliable and clear data. Second, since this study was conducted on a nationally representative sample, the results reflect the overall situation in South Korea and could be used to establish health policy.

In conclusion, our results have public health significance because this research provides insight on preventing dyslipidemia, a high-burden disease, by investigating the relationship between weekend CUS and dyslipidemia. Less than 2 h of weekend CUS was negatively related to dyslipidemia, especially among male workers. Workers with 7–8 h of sleep, night workers, and white-collar workers with CUS were at relatively low risk of dyslipidemia compared to those without CUS. This suggests that properly replenishing sleep on weekends for workers with a lack of sleep on weekdays can help prevent dyslipidemia. Further studies are needed to clarify the neurobiological mechanisms underlying the association of the balance of sleep duration with dyslipidemia.

## Data Availability

The data analyzed in this study were taken from the 2019–2020 KNHANES which is available to the public. All data can be downloaded from the KNHANES official website (https://knhanes.cdc.go.kr/).
