# Author Correction: Association between weekend catch-up sleep and dyslipidemia among Korean workers

**DOI:** 10.1038/s41598-023-29768-6

**Published:** 2023-02-14

**Authors:** Ye Seul Jang, Yu Shin Park, Kyungduk Hurh, Eun-Cheol Park, Sung-In Jang

**Affiliations:** 1 Department of Public Health, Graduate School, Yonsei University, Seoul, Republic of Korea; 2 Institute of Health Services Research, Yonsei University, Seoul, Republic of Korea; 3 Department of Preventive Medicine and Institute of Health Services Research, Yonsei University College of Medicine, 50 Yonsei-Ro, Seodaemun-Gu, Seoul, 03722 Republic of Korea

Correction to: *Scientific Reports* https://doi.org/10.1038/s41598-023-28142-w, published online 17 January 2023

The original version of this Article contained an error in the Acknowledgements section.

“This research did not receive any specific grant from funding agencies in the public, commercial, or not-for-profit sectors.”

now reads:

“This work was supported by the National Research Foundation of Korea (NRF) grant funded by the Korea government (MSIT) (No. 2022R1F1A1062794).”

The original Article has been corrected.

